# Exclusive Breastfeeding and Developmental and Behavioral Status in Early Childhood

**DOI:** 10.3390/nu5114414

**Published:** 2013-11-11

**Authors:** Olof H. Jonsdottir, Inga Thorsdottir, Geir Gunnlaugsson, Mary S. Fewtrell, Patricia L. Hibberd, Ronald E. Kleinman

**Affiliations:** 1 Unit for Nutrition Research, Landspitali—The National University Hospital of Iceland and Faculty of Food Science and Nutrition, School of Health Sciences, University of Iceland, Eiriksgata 29, Reykjavik 101, Iceland; E-Mail: ingathor@hi.is; 2 Directorate of Health and Reykjavik University, Reykjavik 101, Iceland; E-Mail: geirg@hr.is; 3 Childhood Nutrition Research Centre, UCL Institute of Child Health, London WC1N 1EH, UK; E-Mail: m.fewtrell@ucl.ac.uk; 4 Division of Global Health, Massachusetts General Hospital for Children, Harvard Medical School, Boston, MA 02114, USA; E-Mail: phibberd@partners.org; 5 Department of Pediatrics, Massachusetts General Hospital for Children, Harvard Medical School, Boston, MA 02114, USA; E-Mail: rkleinman@partners.org

**Keywords:** early childhood, exclusive breastfeeding, complementary feeding, developmental status, behavior, randomized trial, ISRCTN41946519

## Abstract

Breastfeeding during infancy may have beneficial effects on various developmental outcomes in childhood. In this study, exclusively breastfed infants were randomly assigned to receive complementary foods from the age of 4 months in addition to breast milk (CF, *n* = 60), or to exclusively breastfeed to 6 months (EBF, *n* = 59). At 18 months and again at 30–35 months of age, the children were evaluated with the Parent’s Evaluation of Developmental Status questionnaire (PEDS) and the Brigance Screens-II. The parents completed the PEDS questionnaire at both time intervals and the children underwent the Brigance Screens-II at 30–35 months. At 30–35 months, no significant differences were seen in developmental scores from the Brigance screening test (*p* = 0.82). However, at 30–35 months a smaller percentage of parents in group CF (2%) had concerns about their children’s gross motor development compared to those in group EBF (19%; *p* = 0.01), which remained significant when adjusted for differences in pre-randomization characteristics (*p* = 0.03). No sustained effect of a longer duration of exclusive breastfeeding was seen on selected measures of developmental and behavioral status at 18 months, although at 30–35 months, a smaller percentage of parents of children introduced to complementary foods at four months of age expressed concerns about their gross motor development.

## 1. Introduction

Breastfeeding may have beneficial effects on development in childhood, adolescence and even in adulthood [1,2], although this has not been a consistent finding [3]. Furthermore, some studies indicate that a longer duration of exclusive breastfeeding is important for this positive association with developmental outcomes in childhood, especially for those born small for gestational age [4,5,6]. While most studies have focused on cognitive development, less is known about the impact of breastfeeding and the duration of exclusive breastfeeding on non-cognitive developmental and behavioral status in childhood. Some studies indicate that breastfeeding in general, and also, a longer duration of breastfeeding may be associated with decreased risk of behavioral problems and developmental delays in childhood [7,8,9]; however, findings on this subject are inconsistent. A large breastfeeding promotion intervention in Belarus showed no relationship between prolonged breastfeeding or longer duration of exclusive breastfeeding and childrens’ behavior at 6.5 years of age [10,11]. Other studies have shown that increased duration and exclusivity of breastfeeding may have beneficial effects on language and motor development in childhood [12,13,14,15,16,17,18].

There has been a longstanding debate about the optimal duration of exclusive breastfeeding; whether infants should be exclusively breastfed for 4 or 6 months after birth [19]. The current recommendations of the WHO are that infants should be exclusively breastfed for the first 6 months of life [20] but until May 2001 the WHO recommended exclusive breastfeeding for 4–6 months of age [21]. We have previously reported the results of a parallel group, masked, randomized controlled trial of the effects of exclusive breastfeeding for 4 *vs*. 6 months on growth, body composition, breast-milk intake and iron status of the infant [22,23]. We now report a secondary analysis from this cohort of exclusive breastfeeding infants for 4 *vs.* 6 months on selected measures of development and behavior in early childhood. We hypothesized that infants exclusively breastfed for 6 months would have better outcomes in selected measures of developmental and behavioral status at 18 months and 30–35 months of age than those receiving complementary foods from 4 months in addition to breast milk. 

## 2. Experimental Section

### 2.1. Study Design

As described previously [22,23], between November 2007 and November 2009, a total of 119 mother-infant pairs were recruited at seven health care centers in Iceland where 50% and 35% of mothers exclusively breastfeed through 4 and 5 months of age, respectively [24]. A total of 656 infants were assessed for eligibility in this randomized controlled trial. Eligibility criteria for the study were singleton birth, gestational length ≥37 weeks, exclusively breastfed, infant characterized as healthy: absence of congenital abnormalities or chronic health issues likely to affect growth, development or iron status. Mothers of eligible infants were invited to participate in the study and infants who were still exclusively breastfed and whose parents were willing to participate were enrolled in the study at 4 months of age. Eligible mother-infant pairs were randomly assigned to receive complementary foods from the age of 4 months in addition to breast milk (CF), or to continue being exclusively breastfed to the age of 6 months (EBF). Vitamin D supplements were recommended in both groups. Exclusive breastfeeding was defined as breastfeeding with no additional liquid or solid foods other than vitamins and medications [25]. The use of up to 10 feedings of formula or water during the first 6 months was allowed to avoid having to exclude infants that in fact were otherwise exclusively breastfed.

The study was reviewed and approved by the Data Protection Authority and National Bioethical Committee in Iceland and the Partners Health System IRB, Boston, MA, USA.

### 2.2. Selected Measures of Developmental and Behavioral Status

Children in the present study were assessed both at 18 months and 30–35 months of age, during their routine health care visits at the health center, where developmental and behavioral status was assessed with both the Parent’s Evaluation of Developmental Status (PEDS) questionnaire and the Brigance Screens-II. The parents filled out the PEDS questionnaire at both visits, at 18 months and 30–35 months of age, and the children underwent the Brigance Screens-II at 30–35 months. Both tests were administered by trained nurses at each health care center following prescribed protocols [26,27,28]. PEDS questionnaire and Brigance Screens-II were both introduced in 2010 as part of routine health care visits at health centers in Iceland.

**Figure 1:**
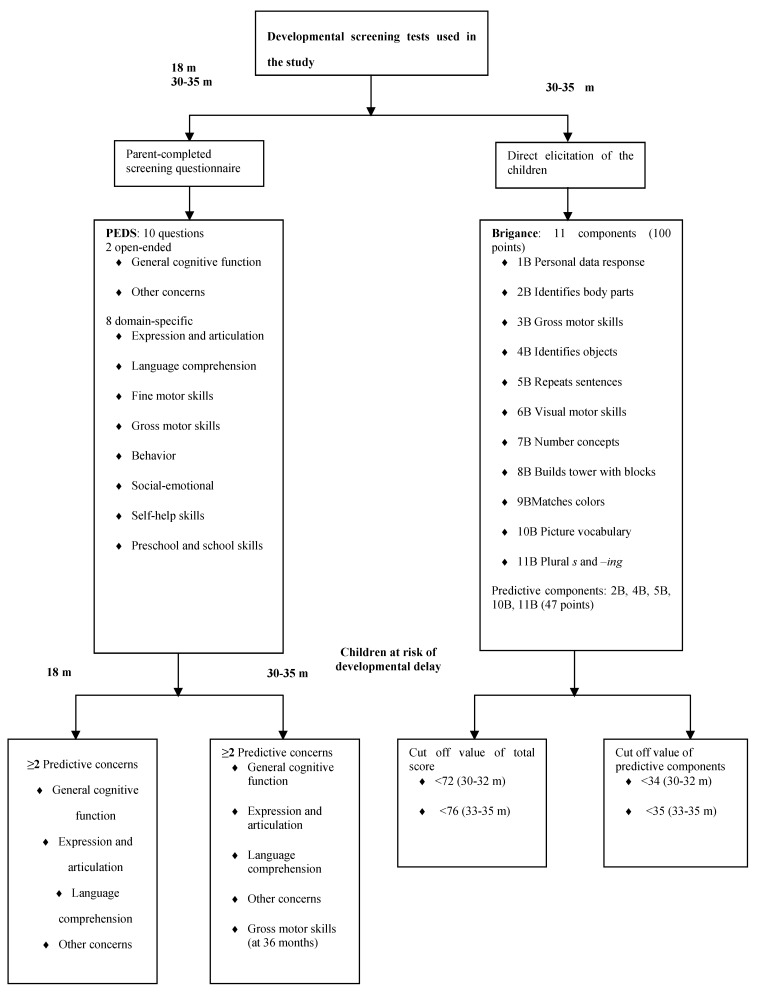
Developmental screening tests used in the study, the Parent’s Evaluation of Developmental Status (PEDS) questionnaire and the Brigance Screens-II.

The PEDS is designed to detect parental concerns about the developmental status and behaviors of their child; it has been found to have very good reliability and has been validated for children from birth to 8 years of age [29,30,31,32,33]. The PEDS questionnaire consists of 10 brief questions, two open-ended about general cognitive function and other concerns and eight domain-specific items. For each of the eight domain-specific questions the parents are asked if they have any concerns about the development or behavior of their child and their response option is in a multiple-choice format (no, yes, a little). Certain parental expressions of concern in response to certain of these questions are predictive of developmental delay [26]. If parents express concern in response to >2 of these predictive questions, then health center procedures require that the child be referred for further evaluation (see Figure 1). It takes parents approximately 5 min to answer the questionnaire. 

The Brigance Screens-II is administered by a trained nurse who observes the child and questions his/her parents and the test is completed by the child itself. It has good reliability and has been validated for measuring the developmental and behavioral status of toddlers and preschool children [34,35,36]. The Brigance Screens-II for 30–35 month old children is valid for children from the age of 29 months + 15 days to 35 months + 14 days old children. The Brigance Screens-II comprises 11 components and it takes children approximately 15–20 min to complete the test. The cut off points for defining children at risk of developmental delay are <72 and <76 points of 100 points for children aged 30–32 months and 33–35 months, respectively. As with the PEDS questionnaire, there are some components of the Brigance Screens-II more predictive for developmental delay than others among all the test components (see Figure 1). Cut off points for defining children at risk of developmental delay are <34 and <35 points for children aged 30–32 and 33–35 months, respectively [37]. In the current study we focused on assessment of gross motor skills (3B), fine motor skills (6B, 8B) and receptive or expressive language (5B, 10B, 11B), since studies indicate that breastfeeding may influence these factors [12,13,14,15,16,17,18].

### 2.3. Statistical Analysis

Data were analyzed with SPSS Windows statistical software package version 20.0 (SPSS Inc., Chicago, IL, USA) with a level of significance of *p* ≤ 0.05. Data were presented with means and standard deviations (SD) for normally distributed variables and with median and interquartile range (IQR) for variables with skewed distribution. Group comparisons were performed using independent-samples *t*-test and Mann-Whitney U-test. Comparisons between categorical values were made using the Chi-square tests of association or two-sided Fisher’s exact test. Regression analysis was performed to adjust for any pre-randomization characteristics that were different between the two intervention groups at baseline. Finally, we calculated the power to detect differences between the CF and EBF groups based on proportions. To detect a significant difference between intervention groups in developmental scores from the Brigance screening test at 30–35 months of age with a sample size of 66 and a power of 80%, the mean difference in developmental scores would have had to be approximately 11.2 points, or approximately 5.4 points if excluding the three outliers (*n* = 63). Of the 100 mother-infant pairs who finished the breastfeeding intervention trial, a total of 95 children attended routine care at 18 months and 82 at 30–35 months. Fifty-four parents answered the PEDS questionnaire when their child was 18 months and 78 parents at the 30–35 months visit. These numbers are based on the calculation of the sample size. 

## 3. Results

### 3.1. Sample Size and Characteristics of Participants

Since both PEDS questionnaire and Brigance Screens-II were introduced in 2010 we have 41 missing data points from PEDS questionnaire at 18 months of age for those children in the study born in 2007 and some who were born in 2008. Parents of 4 children who attended routine care at 30–35 months did not answer the PEDS questionnaire at that age. The Brigance Screens-II was undertaken by 77 children at the age of 30–35 months, but 10 of them were too old (>35 months + 14 days) and 1 too young (<29 months + 15 days) when the Brigance Screens-II was performed and were therefore excluded from the analysis. The PEDS questionnaire is for a wider age range but we chose to use 30–35 months throughout the paper. The children that did not have developmental scores recorded from the Brigance Screens-II (*n* = 23) were lost to follow-up for several reasons, such as the family had moved abroad or failure to attend the routine health care visits at the health center. 

Among those children with developmental scores from the Brigance Screens-II at 30–35 months of age (*n* = 66), no differences between study groups were seen in baseline characteristics, except for mode of delivery, where vaginal delivery was more common among children in the CF group (94% *vs.* 74% in the EBF group, *p* = 0.04) (see Table 1). No difference was seen in baseline characteristics (same as seen in Table 1) among those who were followed-up (*n* = 82) and those who were lost to follow-up (*n* = 18), except for parity, where those parents who were lost to follow-up had more children (3.0 ± 1.0 children) than those who were followed-up (2.0 ± 2.0 children; *p* = 0.01).

**Table 1:** Baseline characteristics of participants with scores from Brigance Screens-II at 30–35 months of age in the two study groups: infants who received complementary foods in addition to breast milk from 4 months (CF, *n* = 35) compared with infants who were exclusively breastfed for 6 months (EBF, *n* = 31).

Variables	Group CF	Group EBF
Boys	17 (49%) *	13 (42%) *
Birth weight (g)	3687 (432)	3733 (526)
Length at birth (cm)	51.3 (1.8)	51.7 (1.9)
Head circumference at birth (cm)	35.8 (1.3) ^†^	35.9 (1.4)
Gain in head circumference from birth–18 months (cm)	12.6 (1.2) ^‡^	12.6 (1.7) ^§^
Age when Brigance Screens-II was performed (months)	32.3 (1.6)	32.8 (1.6)
Gestational length (days)	280.5 (9.3)	280.8 (7.1)
Maternal age (years)	29.4 (4.4)	31.2 (4.8)
Maternal education ^║^	22 (63%) *	16 (52%) *
Vaginal delivery	33 (94%) *	23 (74%) *
Parity	2.0 (2.0) ^¶^	2.0 (1.0) ^¶^
Father’s education ^║^	13 (38%) *^‡^	14 (45%) *

Data are presented as mean (SD) unless otherwise indicated; * Data are presented as number (%); ^†^ One missing value, *n* = 34; ^‡^ Three missing values, n = 32; ^§^ Five missing values, *n* = 26; ^║^ Finished studies at university level; ^¶^ Data are presented as median (IQR).

### 3.2. Developmental and Behavioral Status

Table 2 shows the developmental and behavioral status measures in the two study groups at 18 months (PEDS questionnaire) and at 30–35 months (PEDS questionnaire and Brigance Screens-II). At 18 months, a significantly smaller percentage of parents had concerns about any of the domains of PEDS on their children’s developmental and behavioral status in the CF group compared with those in the EBF group (17% in the CF group *vs.* 44% in the EBF group; *p* = 0.03). A logistic regression was done to test the impact of the intervention by group, and when adjusted for mode of delivery, the difference in parents’ concerns between groups at 18 months was not statistically significant (*p* = 0.08). No difference was seen between groups in the number of concerns regarding gross or fine motor skills or receptive and expressive language. At 18 months, parents most often expressed concerns about their children’s expression and articulation of the eight domain-specific questions; 10% and 20% in the CF and EBF group, respectively (*p* = 0.45). No significant differences were seen even when those questions with greater predictive value for developmental delay were compared among groups at 18 months (0% in the EBF group *vs.* 3% in the CF group; *p* = 1.0).

At 30–35 months of age no significant differences were seen between study groups in number of parents with concerns about any of the domains of PEDS (42% in the EBF group *vs.* 33% in the CF group; *p* = 0.45). A smaller proportion of parents of children in the CF group (2%) had concerns about their gross motor development compared with parents of those in the EBF group (19%; *p* = 0.01). When adjusted for mode of delivery the difference was still significant (*p* = 0.03). No difference was seen between groups in number of concerns regarding fine motor skills or receptive and expressive language. At 30–35 months, parents most often expressed their concerns about their children’s expression and articulation of the eight domain-specific questions; 19% and 28% in the CF and EBF group, respectively (*p* = 0.36). Use of the cut off of ≥2 predictive concerns for PEDS questionnaire at 30–35 months showed that 19% of parents in the EBF group were above the cut off value compared with 5% of the parents in the CF group, although the difference was not significant (*p* = 0.07). 

**Table 2:** Selected measures of developmental and behavioral status for children at 18 months and at 30–35 months of age in the two intervention groups: infants who received complementary foods in addition to breast milk from 4 months (CF) compared with infants who were exclusively breastfed for 6 months (EBF).

Variables	Group CF	Group EBF	P-value
*PEDS questionnaire*			
Parents with concerns according to PEDS at 18 months	5 (17%) *; *n* = 29	11 (44%) *; *n* = 25	0.03
Parents with concerns according to PEDS at 30–35 months	14 (33%) *; *n* = 42	15 (42%) *; *n* = 36	0.45
*Brigance Screens-II*	*n* = 35	*n* = 31	
Total score at 30–35 months	86.0 (12.5) ^†^	86.5 (12.5) ^†^	0.82
Total score above cut off value ^‡^	2 (6%) *	4 (13%) *	0.41
Score of predictive factors combined above cut off value ^§^	7 (20%) *	3 (10%) *^║^	0.32
*Components of the Brigance Screens-II*			
Gross motor skills	6.0 (6.0) ^†^	6.0 (4.5) ^†║^	0.44
Fine motor skills	19.0 (3.0) ^†^	19.0 (3.0) ^†║^	0.89
Expressive and receptive language	40.5 (8.0) ^†^	42.0 (9.5) ^†║^	0.81

Data are presented as mean (SD) unless otherwise indicated; * Data are presented as number (%); ^†^ Data are presented as median (IQR); ^‡^ Cut off values for defining risk of developmental delay were <72 and <76 points from the total score from the Brigance Screens-II for children aged 30–32 months and 33–35 months, respectively; ^§^ Cut off values for defining risk of developmental delay were <34 and <35 points from the predictive components of the Brigance Screens-II combined for children aged 30–32 months and 33–35 months, respectively; ^║^ Two missing values, *n* = 29.

There was no significant difference between study groups at 30–35 months by the Brigance Screens-II (*p* = 0.82). Neither was there a significant difference between the groups in the number of children below the cut off value defining developmental delays for total score from the Brigance Screens-II (*p* = 0.41) or number of children above the cut off value defining developmental delays from predictive components of the Brigance screening test combined (*p* = 0.32). Furthermore, there was no significant difference between groups in fine or gross motor skills or receptive and expressive language according to the Brigance Screens-II at 30–35 months. Excluding three outliers found in the EBF group in the Brigance screening test did not change the mean values for the study groups or the lack of significance (86.1 ± 7.8 points in the CF group *vs.* 88.0 ± 7.4 points in the EBF group; *p* = 0.33). 

## 4. Discussion

In this study of well-nourished children at 30–35 months of age, a smaller proportion of parents in the CF group expressed their concerns about their children’s gross motor development on the PEDS questionnaire, a difference that remained significant when adjusted for differences in pre-randomization characteristics. However, there were no significant intergroup differences in developmental total scores or in fine and gross motor skills or receptive and expressive language according to the Brigance Screens-II at 30–35 months. No difference was seen in the percentage of parents with concerns about their children’s developmental and behavioral status at the age of 18 months.

Results from the PEDS questionnaire are based on a small number of categorical variables. Outcomes from the Brigance Screens-II, however, are based on continuous variables and therefore this test is more responsive to detecting minor developmental disabilities. The Brigance Screens-II is a comprehensive, reliable and valid screening tool of developmental status that is completed by the child itself [36,38]. Similar general developmental screening tools that are directly administered to the child and are used in primary care settings are the Battelle Developmental Inventory Screening Tool Test II, the Bayley Infant Neurodevelopmental Screener and the Denver-II Developmental Screening Test, which are all comparable to the Brigance Screens-II in sensitivity and specificity [38,39]. Per health center protocols in the Icelandic healthcare system, children identified at risk for developmental delay or behavioral problems according to the Brigance Screens-II or the PEDS questionnaire are referred for further evaluation, diagnosis and then developmental intervention, if appropriate. Early detection of developmental delay and appropriate intervention has been shown to be effective in improving developmental outcomes in childhood [40].

Although the PEDS is solely based on parental perception of their children’s developmental and behavioral status, a positive correlation has been shown between the results of the PEDS questionnaire and the Brigance Screens-II [28]. The PEDS questionnaire is a valid and reliable developmental screening tool [38] and the value of parents’ concerns in the detection of developmental delay has been well studied [31,41]. Comparable commonly used parent-completed screening questionnaires in primary care settings comparable are the Ages & Stages Questionnaires, the Child Development Review-Parent Questionnaire and the Infant Development Inventory [38,39]. These tests are not perfectly concordant, but are widely used and are considered appropriate for developmental evaluation in primary care settings [42,43]. It should be noted that although some parental concerns are predictive over time, the PEDS questionnaire does not always capture longitudinal changes in developmental status since parents may have fewer concerns after their child begins a developmental intervention, even though developmental delays may still be present. In 2006, the American Academy of Pediatrics recommended systematic developmental screening in primary care using a validated screening tool for children aged 9, 18 and 30 months, but no specific guidance was provided for the specific screening tools that should be used [38]. Health care centers in Iceland chose to use the PEDS questionnaire and the Brigance screening test because of their good reliability and validity and well-established sensitivity and specificity and because they are useable for a wide range of ages in childhood [28].

To our knowledge this is the first secondary analysis of a randomized controlled trial conducted in a resource rich country to examine the effects of exclusive breastfeeding for 4 *vs.* 6 months on selected measures of developmental and behavioral status in early childhood. The World Health Organization (WHO) recommended exclusive breastfeeding for 4–6 months of life until the year 2001 when the recommendation was changed to breastfeed exclusively for the first 6 months of life in an effort to lower the risk of adverse health outcomes for infants during the first 6 months, particularly in resource constrained countries [44]. Developmental status is influenced by a number of genetic and environmental factors that cause cumulative risk effects of development delays that are generally not addressed in observational studies. This is one possible explanation for the inconsistent findings among such studies [13,45,46]. Studies investigating the relationship between breastfeeding and developmental status often compare formula fed infants to breastfed infants [46,47,48,49], but less is known about the impact of exclusive breastfeeding compared to partial breastfeeding.

There is strong evidence that nutrition early in life can have long-term effects on health and development later in life [50,51,52]. It has been suggested that the concentration of long chain polyunsaturated fatty acids in breast milk may explain the enhanced cognitive outcomes reported in some studies comparing breastfed and formula fed infants [53,54,55,56] and therefore the effect of duration of exclusive breastfeeding on developmental and behavioral status would also be a relevant factor in these outcomes. Since infants exclusively breastfed for 6 months in the present study had significantly higher breast milk intakes at 5.5–6 months of age [22], we hypothesized they would have better developmental and behavioral status in early childhood. However, no intergroup differences in measures of developmental and behavioral status outcomes were observed among those that completed the Brigance Screens-II at 30–35 months of age. The parental impressions from the PEDS questionnaire administered when the children were 30–35 months of age were thus not substantiated by the more objective and reliable Brigance Screens-II at the same age. The reason for no difference in these developmental and behavioral measures might be because both study groups consumed a significant amount of breast milk. While the infants in the EBF group consumed significantly more breast milk than those in the CF group (83 g/day, measured using the stable isotope technique) [22], the amount consumed by the CF group was consistent with the recommendations of the WHO [57]. The mothers who participated in our study were generally well-educated and well-supported, and we cannot generalize our findings to other populations. It is possible that in less well-educated or supported mothers, the introduction of a small amount of CF might result in a greater decrease in breast milk production with more impact on health outcomes, including development.

The strength of the present study lies in the fact that this is the only analysis of later developmental and behavioral data from a randomized controlled trial of 4 *vs.* 6 months of exclusive breastfeeding and it therefore has a methodological advantage over previously published observational studies. Furthermore, approximately 78% of the cohort was follow-up until the age of 30–35 months. The main limitation of the present study is that data were collected in routine health care visits at the health center. We recognize that this secondary data analysis may have been underpowered to detect small effects on developmental and behavioral outcomes that may be biologically relevant, but the sample size was adequate to exclude large effects on developmental and behavioral outcomes in the two groups [58]. 

In addition to breast milk *per se*, other factors that influence infant development may have played a role in the outcomes we observed in this randomized trial. Infants with depleted iron stores, iron deficiency or iron deficiency anemia can have lower developmental scores in childhood [59,60], however, we have previously reported that no differences was seen in the prevalence of iron deficiency with or without anemia between both groups at 6 months of age [23]. Mothers who choose to breastfeed may differ from those who never breastfeed in many ways that can influence an infant’s development, including socio-economic status and nurturing qualities. However, mothers in both study groups exclusively breastfed for the first 4 months of their infant’s life all were from a similar socioeconomic background and thereafter all of them continued breastfeeding partially or exclusively until 6 months of age or beyond, minimizing the impact of these other influential developmental factors. It is possible that the mothers participating in the study might differ from other mothers in the population.

In conclusion, the present study showed no sustained effect of a longer duration of exclusive breastfeeding on selected measures of developmental and behavioral status at 18 months of age although at 30–35 months, a smaller percentage of parents of infants introduced to complementary foods at 4 months of age expressed concerns about their children’s gross motor development. Further investigation is needed in a larger randomized controlled trial using the same or other measures of developmental and behavioral status to extend and confirm these findings. 

## 5. Conclusions

Breastfeeding during infancy may have beneficial effects on various developmental outcomes in childhood. The association between breastfeeding and developmental status is based on observational studies that are subject to bias by confounding factors. In this randomized controlled trial, no sustained difference were seen on selected measures of development and behavior in early childhood between those receiving complementary foods in addition to breast milk from 4 months or those exclusively breastfed for 6 months. Further investigation is needed in a larger randomized controlled trial using the same or other measures of developmental and behavioral status to extend and confirm these findings.
